# HCl-gas etching behavior of (001) β-Ga_2_O_3_ under oxygen supply

**DOI:** 10.1080/14686996.2025.2546285

**Published:** 2025-09-03

**Authors:** Yuichi Oshima, Takayoshi Oshima

**Affiliations:** Research Center for Electronic and Optical Materials, National Institute for Materials Science, Tsukuba, Japan

**Keywords:** β-Ga_2_O_3_, etching, plasma-free, HCl gas, planar etch rate, lateral etch rate

## Abstract

The planar and lateral HCl-gas etching behavior of (001) β-Ga_2_O_3_ under oxygen supply were investigated at partial pressures of *P*^0^(O_2_) = 0–2.5 kPa and 645–1038°C, while maintaining a constant HCl supply partial pressure of *P*^0^(HCl) at 63 Pa. At 747°C, the planar etch rate (PER) exhibited a slight decrease with increasing *P*^0^(O_2_). Notably, at *P*^0^(O_2_) = 1.25 kPa, the PER increased with temperature, demonstrating a plateau between 747 and 848°C, whereas the thermodynamically calculated etching driving force did not. Even minimal O_2_ supply effectively suppressed root mean square (RMS) roughness to <1 nm at 747°C. At *P*^0^(O_2_) = 1.25 kPa, RMS roughness remained at  <2 nm at up to 847°C, but sharply increased to  >7 nm above 947°C, indicating that lower temperatures realize smoother surfaces. Lateral etch rate (LER) analysis, employing a spoke-wheel pattern mask at 747°C revealed significant anisotropy, demonstrating a kidney-like polar plot pattern, with minimum values in the <100 > direction and maximum values in the  <010> direction. Although *P*^0^(O_2_) had a limited effect on anisotropy, temperature increase significantly enhanced the LER, particularly along the ± 20°-rotated directions from  <100> . Above 947°C, etched sidewalls exhibited a multi-faceted morphology owing to the formation of {310} and {3̅10} facets depending on the spoke direction, whereas the sidewalls were relatively smooth below 848°C. These findings underscore the potential of controlled HCl-gas etching for the plasma-free processing of β-Ga_2_O_3_, enabling the fabrication of high-performance devices.

## IMPACT STATEMENT

This study introduces O₂-supplemented plasma-free etching for b-Ga_2_O_3_, achieving enhanced surface smoothness and anisotropic control—providing practical guidelines for 3D structure formation and pre-epitaxial surface preparation.

## GRAPHICAL ABSTRACT

**Figure:**
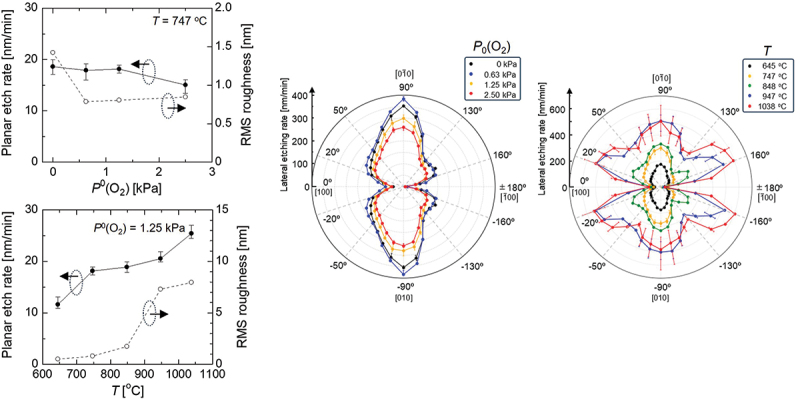

## Introduction

Monoclinic-structured β-Ga_2_O_3_ is an ultrawide-bandgap semiconductor with a bandgap energy ranging from 4.5 to 4.9 eV [1,2]. Among various polymorphs of Ga_2_O_3,_ β-Ga_2_O_3_ is thermodynamically the most stable form, and a bulk single crystal can be produced using melt-growth techniques [3–8]. This availability of high-quality single-crystal β-Ga_2_O_3_ substrates facilitates the epitaxial growth of high-quality β-(Al,Ga)_2_O_3_ films, promoting extensive research and development aimed at the realization of high-performance β-Ga_2_O_3_-based power devices. Promising device prototypes, such as Schottky barrier diodes (SBDs) [9–12], metal-oxide-semiconductor field-effect transistors (MOSFETs) [13–18], and modulation-doped FETs (MODFETs) [19,20], have already been reported in the literature.

To enhance the breakdown voltage of SBDs and enable the normal-off operation of MOSFETs, β-Ga_2_O_3_-based power devices with three-dimensional (3D) surface structures such as trench SBDs [9–11] and FinFETs [14–16,18] have been developed. These 3D structures are typically fabricated using reactive-ion etching techniques; however, such processes often lead to plasma damage that can deteriorate device performance [10,15]. To overcome this limitation, various plasma-free etching techniques have been investigated [21–31]. Furthermore, plasma-free planar etching technology is also crucial as it is expected to eliminate surface damage resulting from processing techniques such as chemical mechanical polishing [32–34] and surface impurities, such as siloxanes [35–37], prior to epitaxial growth. We developed HCl gas etching as a plasma-free etching method and demonstrated the formation of fins and trenches [23,28–30]. This technique can be conducted under atmospheric pressure and is applicable in epitaxial growth reactors such as halide vapor phase epitaxy (HVPE) and metal-organic vapor phase epitaxy systems.

In general, the growth shape and surface morphology of crystals are influenced not only by the surface energy density of the crystal planes but also by various growth conditions such as precursor partial pressure and growth temperature. Currently, there are no reports detailing the influence of the growth conditions on the growth shape in the vapor-phase growth of β-Ga_2_O_3_. However, in the case of the selective area growth of α-Ga_2_O_3_, it has been reported that the morphology of α-Ga_2_O_3_ islands can be effectively controlled by adjusting the partial pressures of precursors, such as GaCl and O_2_, as well as the growth temperature [38]. Etching can be considered as crystal growth with a negative growth rate, and both the shape of the etched dimple and surface morphology should be controllable by adjusting the etching conditions. We have previously reported the effects of substrate temperature (*T)* and the partial pressure of the HCl supply (*P*^0^(HCl)) on the planar etch rate (PER) of (001) β-Ga_2_O_3_, alongside the cross-sectional morphology of the trenches formed along the [010] direction during selective area etching [30]. Additionally, the anisotropy of the lateral etch rate (LER) was investigated; however, this analysis was constrained to a single etching condition [28].

In this study, to further enhance the controllability of the HCl gas etching of β-Ga_2_O_3_ and facilitate the development of high-performance devices, we focused on O_2_ supply as a new parameter for etching control. We systematically investigated the effects of the partial pressure of O_2_ supply *P*^0^(O_2_) and substrate temperature (*T*) under O_2_ supply on the planar and lateral etching behavior of (001) β-Ga_2_O_3_. Thermodynamic analysis was performed to explain its characteristic etching behavior.

## Experimental methods

HCl gas etching was performed in a lab-made hot-wall quartz tube reactor under atmospheric pressure, with the temperature maintained at *T* = 645–1038°C for 10 min. HCl gas ( >99.999% purity) and O_2_ gas ( >99.99995% purity) were supplied at *P*^0^(HCl) = 63 Pa and *P*^0^(O_2_) = 0–2.50 kPa, respectively. The value of *P*^*0*^(HCl) was selected because it was mainly employed in our previous studies [28,30], thereby facilitating direct comparison with those reports. N_2_ (dew point <−110 °C) was utilized as the carrier gas.

Single-crystal (001) β-Ga_2_O_3_ substrates (Novel Crystal Technology, Inc.) were employed for the etching experiments. A β-Ga_2_O_3_ substrate was covered with a SiO_2_ mask that featured a window of 3 × 3 mm^2^ to investigate the PER for the (001) plane. The PER was calculated from the depth of the etched dimple at the window, measured using a stylus profiler (Dektak XT-A, Bruker, USA) after the removal of the mask with buffered hydrofluoric acid at room temperature. Atomic force microscopy (AFM) was performed using Jupiter XR (Oxford instruments, UK) to observe the morphology of the etched surfaces and to determine the root mean square (RMS) roughness.

**Figure 1.**
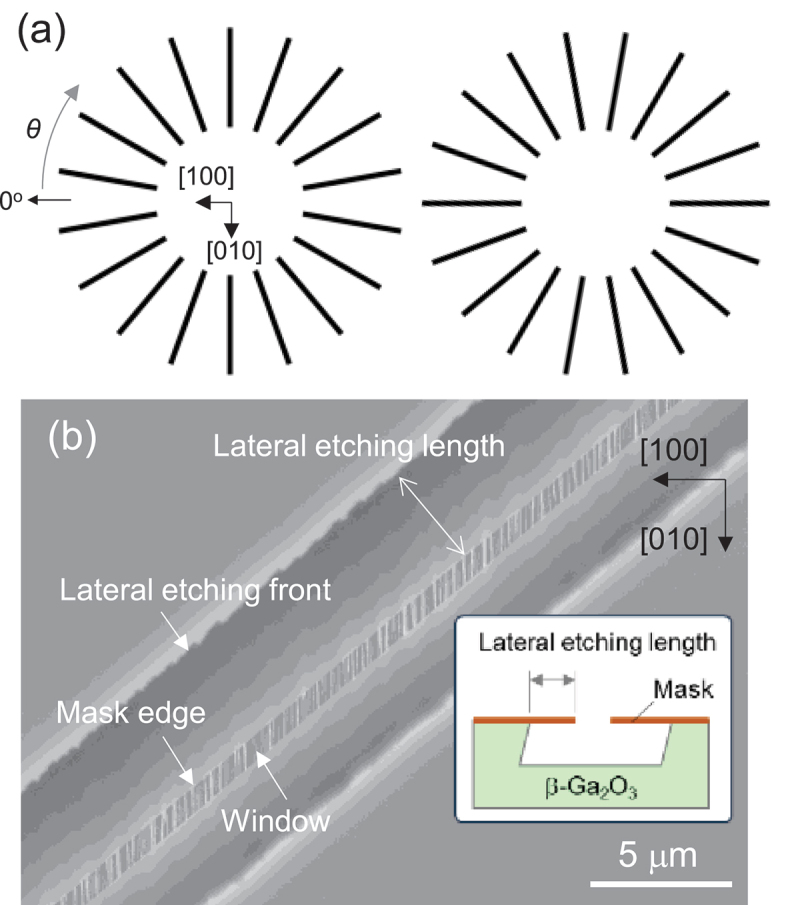
(a) Schematic of the spoke-wheel pattern used for the LER measurements and the definition of the in-plane angle *θ*. Black lines show the windows. (b) Plan-view SEM image of a spoke after etching. (inset) a schematic showing the definition of a lateral etching length.

To examine the LER, a β-Ga_2_O_3_ substrate was covered with a SiO_2_ mask featuring a spoke-wheel pattern, comprising rectangular windows of 0.6 × 30 μm^2^ and aligned in 20° steps. The in-plane direction (*θ*) was defined to increase in a clockwise manner in the 0° direction along the [100] direction, as illustrated in Figure 1(a). Two adjacent spoke-wheel patterns with the initial spoke angles of 0° and 10° were utilized for this analysis (Figure 1(a)). The LER was quantified by measuring the lateral etching length, defined as the distance between the mask edge and etching front, and was analyzed through field-emission scanning electron microscopy (FE-SEM) using SU8230 (Hitachi, Japan) (Figure 1(b)). A high acceleration voltage of 20 kV was employed to observe the etching front through the SiO_2_ mask.

## Results and discussion

**Figure 2.**
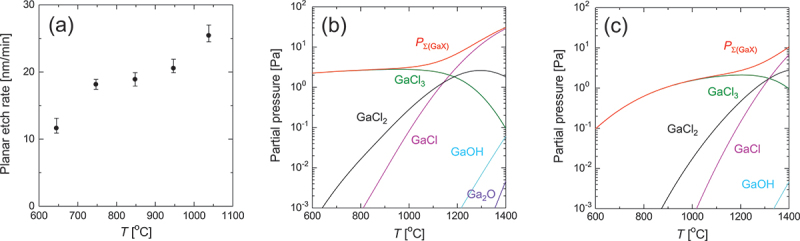
(a) PER of (001) β-Ga_2_O_3_ as a function of *T* under a gas supply condition of *P*^0^(HCl)/*P*^0^(O_2_) = 63 Pa/1.25 kPa. (b), (c) thermodynamically calculated equilibrium partial pressures of Ga-containing gaseous species and *P*_Σ(GaX)_ as a function of *T* under the HCl supply condition of *P*^0^(HCl) = 63 Pa and oxygen supply conditions of (b) *P*^0^(O_2_) = 0 and (c) 1.25 kPa.

Figure 2(a) illustrates the temperature dependence of the PER on the (001) plane at *P*^0^(HCl)/*P*^0^(O_2_) = 63 Pa/1.25 kPa. It is revealed that the PER increased with the temperature and exhibited a plateau at *T* = 747–848°C, which was not observed in the absence of O_2_. The appearance of a plateau due to O_2_ addition aligns with previously reported results for the etching of (010) β-Ga_2_O_3_ using HCl gas produced via the thermal decomposition of tert-butyl chloride (TBCl) within a similar temperature range [27].

To elucidate the cause of the plateau, the effect of O_2_ supply on the temperature dependence of the etching driving force was investigated through thermodynamic analysis. Upon etching β-Ga_2_O_3_, volatile species containing Ga should be generated. The etching driving force of β-Ga_2_O_3_ is expected to be proportional to the following quantity *P*_Σ(GaX)_, where *P*(Ga_*n*_X) represents the equilibrium partial pressure of the Ga-containing gaseous species Ga_*n*_X (where *n* represents the number of Ga atoms in the molecule), on the β-Ga_2_O_3_ surface. Note that *P*_Σ(GaX)_ does not include the partial pressure of the carrier gas.

(1) $$P_{\Sigma \left(\mathrm{GaX}\right)}=\underset{i}{\sum }n_{i}P_{i}\left(Ga_{n_{i}}X_{i}\right)$$

Accordingly, the dependence of *P*_Σ(GaX)_ on *T* and *P*^0^(O_2_) was theoretically calculated using Equation (1). The equilibrium partial pressure of each gaseous species was determined using a thermodynamic analysis software (CatCalc, Research Institute of Computational Thermodynamics, Inc.). This software calculates the equilibrium partial pressures of the gaseous species to minimize the Gibbs energy of a system [39]. The total pressure of the system and *P*^0^(HCl) were set to 100 kPa and 63 Pa, respectively, with *P*^0^(O_2_) varying between 0 and 1.25 kPa. N_2_ was served as the carrier gas. In these calculations, the Ga-containing gaseous species generated during etching were assumed to include GaCl_*n*_, (GaCl_*n*_)_2_ (*n* = 1–3), GaOH, GaO, Ga_2_O, GaH, Ga, and Ga_2_. In addition, the Ga-free gaseous species considered in this calculation, excluding the carrier gas, include H_2_, HCl, HClO, HClO_4_, H_2_O, H_2_O_2_, O_2_, O_3_, Cl_2_, ClO_2_, and Cl_2_O. It should be noted that in the etching of Ga_2_O_3_, not only externally supplied HCl but also gaseous species such as H₂–formed as etching byproducts – may contribute secondarily. Since the thermodynamic calculations in this study include such byproducts in the equilibrium calculations, the results presented here should inherently reflect those secondary etching effects.

Figure 2(b) presents the calculated results for the case without O_2_ supply, revealing that GaCl_*n*_ should emerge as the primary etching product. The equilibrium partial pressures of other Ga-containing species are outside the plotting range ( <10^−3^ Pa). Notably, GaCl_3_ dominated as the etching product from low temperatures up to approximately 1000°C. Although *P*(GaCl_3_) decreased at higher temperatures, this reduction was compensated by the increases in *P*(GaCl) and *P*(GaCl_2_), resulting in a continuous rise in *P*_Σ(GaX)_ with temperature. When O_2_ was supplied, as shown in Figure 2(c), the decrease in *P*(GaCl_3_) and the onset of *P*(GaCl) and *P*(GaCl_2_) were significantly shifted to higher temperatures. As a result, the overall etching driving force decreased. This can be interpreted as being due to the contribution of reverse reactions to produce Ga_2_O_3_, such as those between GaCl_*n*_ and O_2_. Note that the experimentally observed plateau was not reproduced. Accordingly, contributions from mechanisms not considered in the thermodynamic analyses, such as the surface microstructure of the crystal and surface coverage by etchants and etching products, are likely responsible for this phenomenon. For instance, in the context of GaAs etching by Cl_2_, variation in the surface Cl coverage induced by temperature can lead to complex, non-Arrhenius behavior in the temperature dependence of the etch rate [40]. Similarly, in the etching of (010) β-Ga_2_O_3_ using HCl derived from the thermal decomposition of TBCl, it has been proposed that temperature-dependent desorption behavior of the etching products may affect the temperature dependence of the etch rate [27]. Further studies are required to elucidate the mechanisms observed in the present study.

**Figure 3.**
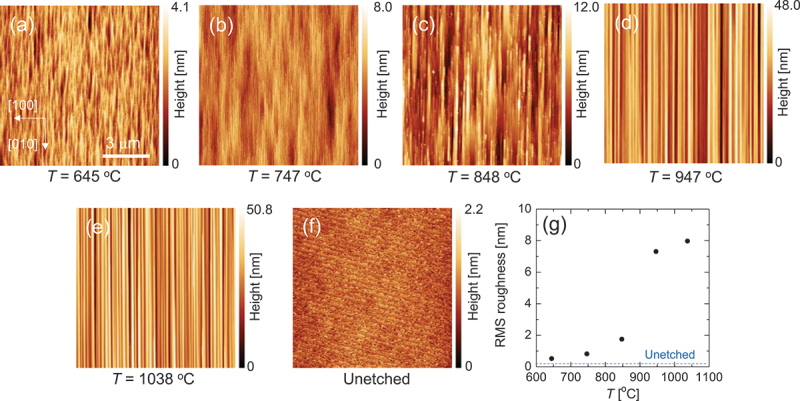
(a)–(e) AFM images of the etched (001) surfaces under a gas supply condition of *P*^0^(HCl)/*P*^0^(O_2_) = 63 Pa/1.25 kPa. (f) AFM image of an unetched substrate. (g) RMS roughness of the etched surface as a function of *T*.

Figure 3(a–e) illustrate the AFM images depicting temperature-dependent changes in the surface morphology after planar etching. For comparative analysis, an AFM image of the unetched sample is displayed in Figure 3(f). The unetched surface demonstrated atomic steps corresponding to the miscut direction, with an RMS roughness of ∼0.2 nm. In contrast, the etched surfaces demonstrated the gradual development of linear morphologies extending along the *b*-axis with increasing temperature, becoming particularly pronounced at temperatures above 947°C. This temperature aligns closely with the high-temperature threshold of the plateau, as depicted in Figure 2(a), where the PER begins to increase sharply. The distinctive linear morphology at elevated temperatures likely arises from the rapid disappearance of surfaces with lower etching resistance, as the etching driving force intensifies, leaving behind relatively stable crystal planes. Figure 3(g) illustrates the temperature dependence of the RMS roughness of the etched surfaces. With increasing temperature, RMS roughness increased gradually up to 848°C but exhibited a steep increase above 848°C, where the morphology underwent a drastic transformation. Therefore, it was found that lower temperatures were more favorable for obtaining smoother etched surfaces.

**Figure 4.**
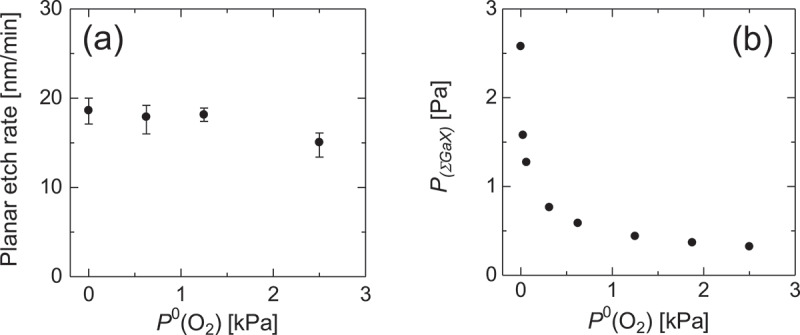
(a) PER of the (001) surface as a function of *P*^0^(O_2_) at *T* = 747°C with the HCl supply of *P*^0^(HCl) = 63 Pa. (b) Calculated *P*_Σ(GaX)_ as a function of *P*^0^(O_2_) assuming the same etching conditions as those in (a).

Figure 4(a) illustrates the PER as a function of *P*^0^(O_2_) at *T* = 747°C with the HCl gas supply maintained at *P*^0^(HCl) = 63 Pa. The PER exhibited a gradual decline with rising *P*^0^(O_2_). Conversely, thermodynamic analysis under identical experimental conditions predicted an initial sharp decrease in *P*_Σ(GaX)_ with the addition of a small amount of O_2_, followed by a gradual decline (Figure 4(b)). The initial sharp decrease, anticipated by calculations, was not evident in the experimental results.

The cause of this discrepancy remains unclear; however, it may be due to the kinetic effects mentioned above, which were not considered in the thermodynamic analyses. Notably, the absolute value of the PER was significantly lower than previously reported [30] (a comparison can be made for the PER at *P*^0^(O_2_) = 0 kPa). This discrepancy may be attributed to the fact that the previous study measured the PER using a much smaller 100 × 100 μm^2^ SiO_2_ mask window than that utilized in the present study. In other words, because the HCl etchant was only consumed within the window region, in the case of smaller windows, the unconsumed etchant on the mask may have contributed to etching within the window region to increase effective *P*^0^(HCl), thereby leading to a higher PER reported in the previous study. Fig. S1 presents an etching depth profile used for calculating the PER. Pronounced etching is observed near the edge of the window area, supporting the presence of the mechanism discussed here. Therefore, when applying the proposed etching technique to the formation of 3D structures in β-Ga_2_O_3_, it is crucial to evaluate the etch rate under realistic window-size conditions. It should be noted that, in this study, the etching depth was measured at locations sufficiently distant from the edges of the window.

**Figure 5.**
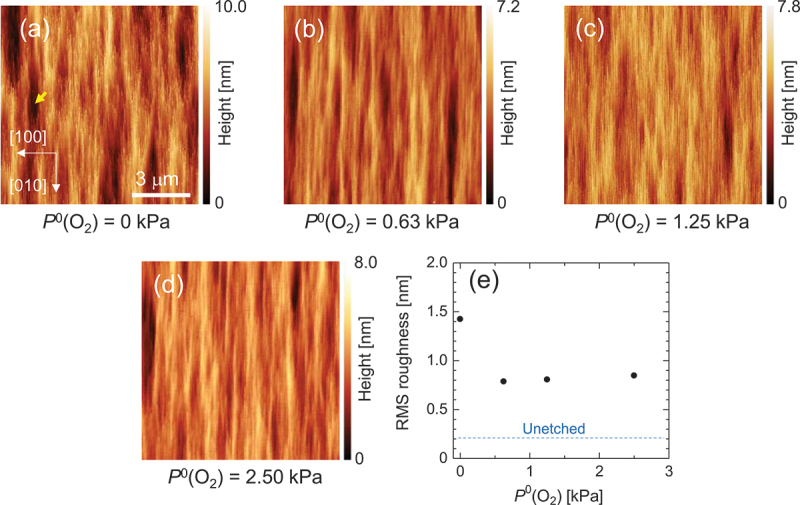
(a)–(d) AFM images of the (001) surface etched at various *P*^0^(O_2_) values and at *T* = 747°C with HCl supply of *P*^0^(HCl) = 63 Pa. (e) RMS roughness of the etched surface as a function of *P*^0^(O_2_).

Figure 5(a–d) present the AFM images that exhibit differences in surface morphology following etching under various *P*^0^(O_2_) conditions. In the absence of O_2_, deep pits, indicated by an arrow for example in Figure 5(a), were observed at relatively high density. In contrast, under O_2_ supply, even at the minimum partial pressure of 50 kPa, the density of the deep pits was significantly reduced. A further increase in *P*^0^(O_2_) did not yield a substantial additional reduction in pit density. This suggests the presence of crystal defects at the center of the pits, indicating that the HCl gas etching may be defect sensitive in the absence of O_2_. Conversely, under O_2_ supply, the reverse reaction of etching, i.e. the formation of β-Ga_2_O_3_ through the reaction between GaCl_*n*_ and O_2_, likely occurs concurrently. If the formation of β-Ga_2_O_3_ is accelerated around the defects for any reason, the formation of pits may be suppressed. For instance, in the case of dislocations with a screw component, the atomic step density near the dislocation may be elevated, potentially enhancing the growth rate. Figure 5(e) depicts the dependence of RMS roughness on *P*^0^(O_2_). Notably, under O_2_ supply, the RMS roughness significantly decreases, likely correlating with the observed reduction of pit density.

**Figure 6.**
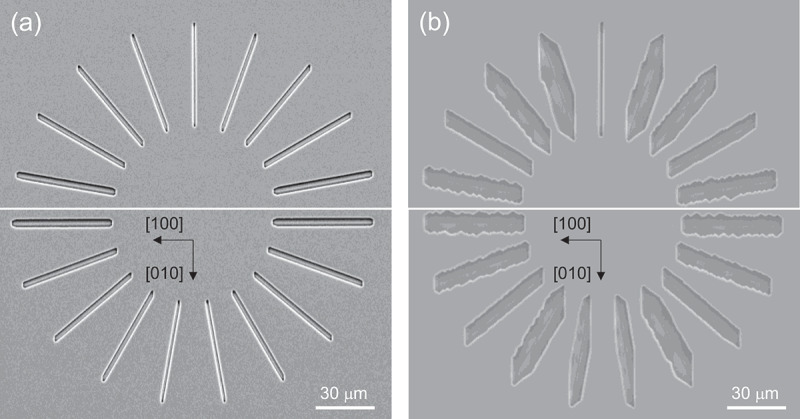
Plan-view SEM images of the samples etched through the spoke-wheel-patterned mask under gas supply conditions of *P*^0^(HCl)/*P*^0^(O_2_) = 63 Pa/1.25 kPa at (a) 645°C and (b) 1038°C.

Figure 6(a,b) present the plan-view SEM images of the spoke-wheel pattern etched at 645 and 1038°C, respectively, under the conditions of *P*^0^(HCl)/*P*^0^(O_2_) = 63 Pa/1.25 kPa. Notably, the LER exhibits an anisotropic increase with rising temperature. Additionally, at elevated temperatures, the spokes that are oriented close to the *a*-axis tend to develop zigzag (multi-faceted with {310} and {3̅10} planes) sidewalls These planes are slip planes associated with the densely packed arrangement of the oxygen sublattice [41], and are considered to be relatively stable crystallographic planes of β-Ga_2_O_3_.

**Figure 7.**
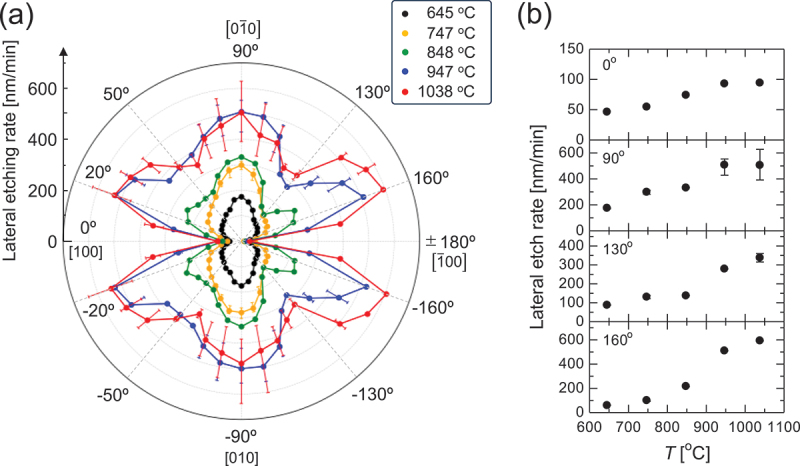
(a) Polar plot of the LER under gas supply conditions of *P*^0^(HCl)/*P*^0^(O_2_) = 63 Pa/1.25 kPa. (b) LER as a function of *T* along selected directions.

Figure 7(a) illustrates a polar plot of the LER under gas supply conditions of *P*^0^(HCl)/*P*^0^(O_2_) = 63 Pa/1.25 kPa. In the <100 > direction (*θ* = 0° and ± 180°), which corresponds to the etching of the {100} plane, which is the strongest cleavage plane with the lowest surface energy density among the crystal planes of β-Ga_2_O_3_ [42], the LER was the smallest in plane, and its increase with temperature was less pronounced compared to that in other directions. Conversely, in the < 010 > direction (*θ* = ±90°), corresponding to the etching of the {010} plane, the LER significantly increased at elevated temperatures. The large error bars at high temperatures indicate the development of zigzag sidewalls, as illustrated in Figure 6(b). The formation of dips around *θ* = ±50° and ± 130° is attributed to these directions correlating with the etching of relatively stable {310} and {3̅10} facets, respectively. The most pronounced increase in the LER with temperature was observed at *θ* ≈ ±20° and ± 160°. Figure 7(b) depicts the temperature dependence of the LER for the discussed representative directions. For *θ* = 90° and 130°, a plateau similar to that observed for the temperature dependence of the PER for the *c*-plane (Figure 2(a)) was observed, likely due to a similar underlying mechanism. In contrast, no plateaus were observed at *θ* = 0° and 160°, suggesting that the presence or absence of a plateau depends on the microstructure of the etched crystal plane and the resulting kinetics.

**Figure 8.**
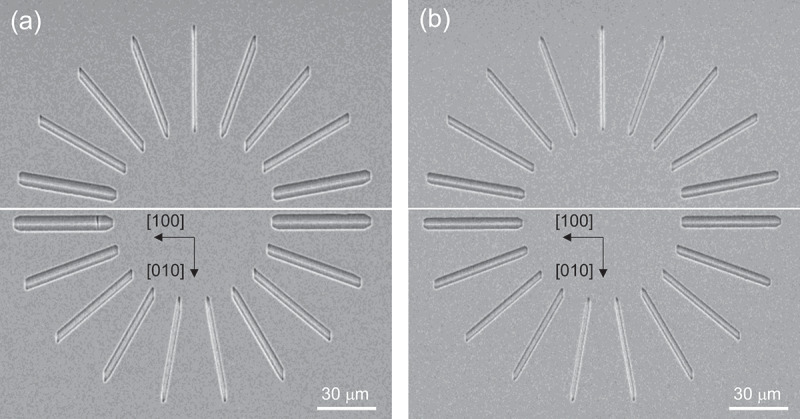
Plan-view SEM images of the samples etched through the spoke-wheel-patterned mask under the conditions of *P*^0^(HCl) = 63 Pa and *T* = 747°C with the *P*^0^(O_2_) of (a) 0 kPa and (b) 2.50 kPa.

Figure 8(a,b) display the plan-view SEM images of the spoke-wheel pattern etched at *P*^0^(O_2_) = 0 and 2.5 kPa, respectively, under the conditions of *P*^0^(HCl) = 63 Pa and *T* = 747°C. The dependence of the LER on *P*^0^(O_2_) was moderate, and no drastic changes in the in-plane anisotropy were observed.

**Figure 9.**
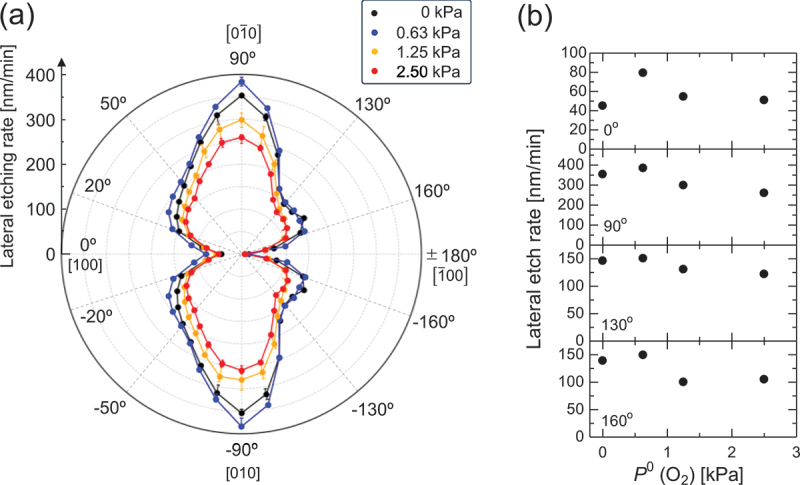
(a) Polar plot of the LER under the conditions of *P*^0^(HCl) = 63 Pa and *T* = 747 °C. (b) LER as a function of *P*^0^(O_2_) along the selected directions.

Figure 9(a) presents a polar plot of the LER of the samples etched under varying *P*^0^(O_2_) conditions at *P*^0^(HCl) = 63 Pa and *T* = 747°C. Regardless of the changes in *P*^0^(O_2_), the LER remains minimized in the < 100 > direction (*θ* = 0° and ± 180°), corresponding to the etching of the most stable {100} plane. In the < 010 > direction (*θ* = ±90°), the LER exhibits the largest increase, accompanied by the development of uneven surfaces on the sidewalls due to the formation of {310} and {3̅10} facets. Dips were observed around *θ* = ±50° and ± 130° (albeit faintly at *θ* = ±50°), becoming more pronounced at lower *P*^0^(O_2_). A notable increase in the LER was also observed at *θ* ≈ ±160°. Figure 9(b) illustrates the temperature dependence of the LER for the representative directions discussed above. In all directions, as *P*^0^(O_2_) was reduced from 2.5 kPa, the LER increased, as expected. However, when no O_2_ was supplied, an unexpected decrease in the LER was observed. This anomalous behavior was not observed in the *P*^0^(O_2_) dependence of the PER for the *c*-plane. The cause of this peculiar behavior remains unclear and cannot be explained thermodynamically; it is speculated to arise from kinetic mechanisms; however, further investigation is required to elucidate the underlying mechanism.

## Conclusions

The effects of *P*^0^(O_2_) and *T* on the etch rate, in-plane etching anisotropy, and RMS roughness of the etched surfaces were investigated for the planar and lateral etching of (001) β-Ga_2_O_3_ using HCl. Notably, the temperature dependence of the PER under O_2_ supply exhibited a plateau, which is believed to be attributable to a specific surface kinetic mechanism. Furthermore, the PER did not exhibit an initial sharp decrease, predicted by thermodynamic analysis with increasing *P*^0^(O_2_); instead, it exhibited a gradual decrease. Additionally, the dependence of the RMS roughness on *P*^0^(O_2_) and *T* was examined, revealing that etching at lower temperatures under O_2_ supply is crucial for achieving smooth etched surfaces. Variations in *P*^0^(O_2_) had a minimal impact on LER or its anisotropy. In contrast, the LER increased substantially with an increase in temperature and exhibited drastic changes in the in-plane anisotropy. Specifically, near the < 010 > direction (*θ* = ±90°) and at *θ* ≈ ±20° and ± 160°, the LER increased significantly, with sidewalls becoming increasingly susceptible to irregularities at temperatures exceeding 947°C. These findings underscore the importance of etching at lower temperatures to achieve smooth sidewalls through lateral etching. However, even at lower temperatures, the sidewalls tended to exhibit a zigzag pattern in directions close to < 010 > . We believe that these findings enhance the versatility and controllability of HCl gas etching and contribute to the development of high-performance β-Ga_2_O_3_-based power devices through the fabrication of 3D structures with high-quality plasma-damage-free surfaces although demonstrating the impact of this etching technique on device performance remains an important subject for future investigation.

## Supplementary Material

Supplemental Material

## References

[1] Orita M, Ohta H, Hirano M, et al. Deep-ultraviolet transparent conductive β-Ga_2_O_3_ thin films. Appl Phys Lett. 2000;77(25):4166. doi: 10.1063/1.1330559

[2] Onuma T, Saito S, Sasaki K, et al. Valence band ordering in β-Ga_2_O_3_ studied by polarized transmittance and reflectance spectroscopy. Jpn J Appl Phys. 2015;54(11):112601. doi: 10.7567/JJAP.54.112601

[3] Aida H, Nishiguchi K, Takeda H, et al. Growth of β-Ga_2_O_3_ single crystals by the edge-defined, film fed growth method. Jpn J Appl Phys. 2008;47(11R):8506. doi: 10.1143/JJAP.47.8506

[4] Blevins JD, Stevens K, Lindsey A, et al. Development of large diameter semi-insulating gallium oxide (Ga_2_O_3_) substrates. IEEE Trans Semicond Manuf. 2019;32(4):466. doi: 10.1109/TSM.2019.2944526

[5] Hoshikawa K, Kobayashi T, Matsuki Y, et al. 2-inch diameter (1 0 0) β- Ga_2_O_3_ crystal growth by the vertical Bridgman technique in a resistance heating furnace in ambient air. J Cryst Growth. 2020;545:125724. doi: 10.1016/j.jcrysgro.2020.125724

[6] Hoshikawa K, Kobayashi T, Ohba E, et al. 50 mm diameter Sn-doped (0 0 1) β- Ga_2_O_3_ crystal growth using the vertical bridgeman technique in ambient air. J Cryst Growth. 2020;546:125778. doi: 10.1016/j.jcrysgro.2020.125778

[7] Galazka Z. Growth of bulk β-Ga_2_O_3_ single crystals by the Czochralski method. J Appl Phys. 2022;131(3):031103. doi: 10.1063/5.0076962

[8] Ueda Y, Igarashi T, Koshi K, et al. Two-inch Fe-doped β-Ga_2_O_3_ (010) substrates prepared using vertical Bridgman method. Jpn J Appl Phys. 2023;62(SF):SF1006. doi: 10.35848/1347-4065/acb55a

[9] Sasaki K, Wakimoto D, Thieu QT, et al. First demonstration of Ga_2_O_3_ trench MOS-type schottky barrier diodes. IEEE Electron Device Lett. 2017;38(6):783. doi: 10.1109/LED.2017.2696986

[10] Li W, Nomoto K, Hu Z, et al. Field-plated Ga_2_O_3_ trench Schottky barrier diodes with a BV^2^/R_on,sp_ of up to 0.95 GW/cm^2^. IEEE Electron Device Lett. 2020;41(1):107. doi: 10.1109/LED.2019.2953559

[11] Otsuka F, Miyamoto H, Takatsuka A, et al. Large-size (1.7 × 1.7 mm^2^) β-Ga_2_O_3_ field-plated trench MOS-type schottky barrier diodes with 1.2 kV breakdown voltage and 10^9^ high on/off current ratio. Appl Phys Express. 2022;15(1):016501. doi: 10.35848/1882-0786/ac4080

[12] Dong P, Zhang J, Yan Q, et al. 6 kV/3.4 mΩ·cm^2^ vertical β-Ga_2_O_3_ schottky barrier diode with BV^2^/R_on,sp_ performance exceeding 1-D unipolar limit of GaN and SiC. IEEE Electron Device Lett. 2022;43(5):765. doi: 10.1109/LED.2022.3160366

[13] Higashiwaki M, Sasaki K, Kamimura T, et al. Depletion-mode Ga_2_O_3_ metal-oxide-semiconductor field-effect transistors on β-Ga_2_O_3_ (010) substrates and temperature dependence of their device characteristics. Appl Phys Lett. 2013;103(12):123511. doi: 10.1063/1.4821858

[14] Chabak KD, Moser N, Green AJ, et al. Enhancement-mode Ga_2_O_3_ wrap-gate fin field-effect transistors on native (100) β-Ga_2_O_3_ substrate with high breakdown voltage. Appl Phys Lett. 2016;109(21):213501. doi: 10.1063/1.4967931

[15] Hu Z, Nomoto K, Li W, et al. Breakdown mechanism in 1 kA/cm^2^ and 960 V E-mode β- Ga_2_O_3_ vertical transistors. Appl Phys Lett. 2018;113(12):122103. doi: 10.1063/1.5038105

[16] Li W, Nomoto K, Hu Z, et al. Single and multi-fin normally-off Ga_2_O_3_ vertical transistors with a breakdown voltage over 2.6 kV. Tech dig-Int Electron Devices Meet. 2019:2019–Decem 270. doi: 10.1109/IEDM19573.2019.8993526

[17] Zeng K, Soman R, Bian Z, et al. Vertical Ga_2_O_3_ MOSFET with magnesium diffused current blocking layer. IEEE Electron Device Lett. 2022;43(9):1527. doi: 10.1109/LED.2022.3196035

[18] Huang HC, Ren Z, Anhar Uddin Bhuiyan AFM, et al. β-Ga_2_O_3_ FinFETs with ultra-low hysteresis by plasma-free metal-assisted chemical etching. Appl Phys Lett. 2022;121(5):052102. doi: 10.1063/5.0096490

[19] Ahmadi E, Koksaldi OS, Zheng X, et al. Demonstration of β-(Al*_x_*Ga _1−*x*_)_2_O_3_/β-Ga_2_O_3_ modulation doped field-effect transistors with Ge as dopant grown via plasma-assisted molecular beam epitaxy. Appl Phys Express. 2017;10(7):071101. doi: 10.7567/APEX.10.071101

[20] Vaidya A, Saha CN, Singisetti U. Enhancement mode β-(Al*_x_*Ga _1−*x*_)_2_O_3_/β-Ga_2_O_3_ heterostructure FET (HFET) with high transconductance and cutoff frequency. IEEE Electron Device Lett. 2021;42(10):1444. doi: 10.1109/LED.2021.3104256

[21] Oshima Y, Ahmadi E, Kaun S, et al. Growth and etching characteristics of (001) β-Ga_2_O_3_ by plasma-assisted molecular beam epitaxy. Semicond Sci Technol. 2018;33(1):015013. doi: 10.1088/1361-6641/aa9c4d

[22] Zhang Y, Mauze A, Speck JS. Anisotropic etching of β-Ga_2_O_3_ using hot phosphoric acid. Appl Phys Lett. 2019;115(1):013501. doi: 10.1063/1.5093188

[23] Oshima T, Oshima Y. Using selective-area growth and selective-area etching on (−102) β-Ga_2_O_3_ substrates to fabricate plasma-damage-free vertical fins and trenches. Appl Phys Lett. 2024;124(4):042110. doi: 10.1063/5.0186319

[24] Huang HC, Kim M, Zhan X, et al. High aspect ratio β-Ga_2_O_3_ fin arrays with low-interface charge density by inverse metal-assisted chemical etching. ACS Nano. 2019;13(8):8784. doi: 10.1021/acsnano.9b01709 31244033

[25] Kalarickal NK, Fiedler A, Dhara S, et al. Planar and three-dimensional damage-free etching of β-Ga_2_O_3_ using atomic gallium flux. Appl Phys Lett. 2021;119(12):123503. doi: 10.1063/5.0057203

[26] Katta A, Alema F, Brand W. Demonstration of MOCVD based in situ etching of β-Ga_2_O_3_ using TEGa. J Appl Phys. 2024;135(7):075705. doi: 10.1063/5.0195361

[27] Gorsak CA, Bowman HJ, Gann KR, et al. In situ etching of β-Ga_2_O_3_ using tert-butyl chloride in an MOCVD system. Appl Phys Lett. 2024;125(24):242103. doi: 10.1063/5.0239152

[28] Oshima T, Oshima Y. Plasma-free dry etching of (001) β-Ga_2_O_3_ substrates by HCl gas. Appl Phys Lett. 2023;122(16):162102. doi: 10.1063/5.0138736

[29] Oshima T, Oshima Y. Anisotropic non-plasma HCl gas etching of a (010) β-Ga_2_O_3_ substrate. Appl Phys Express. 2023;16(6):66501. doi: 10.35848/1882-0786/acdbb7

[30] Oshima Y, Oshima T. Effect of the temperature and HCl partial pressure on selective-area gas etching of (001) β-Ga_2_O_3_. Jpn J Appl Phys. 2023;62(8):80901. doi: 10.35848/1347-4065/acee3b

[31] Oshima T, Togashi R, Oshima Y. Plasma-free anisotropic selective-area etching of β-Ga_2_O_3_ using forming gas under atmospheric pressure. Sci Technol Adv Mater. 2024;25(1):2378683. doi: 10.1080/14686996.2024.2378683 39081843 PMC11288204

[32] Liao ME, Huynh K, Matto L, et al. Optimization of chemical mechanical polishing of (010) β-Ga_2_O_3_. J Vac Sci Technol A. 2023;41(1):013205. doi: 10.1116/6.0002241

[33] Hou T, Ma X, Dong Y, et al. Subsurface damage evolution of β-Ga_2_O_3_ (010) substrates during lapping and chemical Mechanical polishing. Surf Interface. 2024:51 104655. doi: 10.1016/j.surfin.2024.104655

[34] Lavelle RM, Everson WJ, Erdely DJ, et al. Chemical-Mechanical polishing improvements and subsurface damage elimination for Cz grown (010) β-Ga_2_O_3_ substrates. Mat Sci Semicon Proc. 2025;190:109341. doi: 10.1016/j.mssp.2025.109341

[35] Wong MH, Sasaki K, Kuramata A, et al. Electron channel mobility in silicon-doped Ga_2_O_3_ MOSFETs with a resistive buffer layer. Jpn J Appl Phys. 2016;55(12):1202B9. doi: 10.7567/JJAP.55.1202B9

[36] Kumar S, Kamimura T, Lin C-H, et al. Reduction in leakage current through interface between Ga_2_O_3_ epitaxial layer and substrate by ion implantation doping of compensating impurities. Appl Phys Lett. 2020;117(19):193502. doi: 10.1063/5.0029286

[37] McCandless JP, Gorsak CA, Protasenko V, et al. Accumulation and removal of Si impurities on β-Ga_2_O_3_ arising from ambient air exposure. Appl Phys Lett. 2024;124(11):111601. doi: 10.1063/5.0191280

[38] Oshima Y, Kawara K, Oshima T, et al. Rapid growth of α-Ga_2_O_3_ by HCl-boosted halide vapor phase epitaxy and effect of precursor supply conditions on crystal properties. Semicond Sci Technol. 2020;35(5):55022. doi: 10.1088/1361-6641/ab7843

[39] Shobu K. CaTCalc: new thermodynamic equilibrium calculation software. Calphad: Comput Coupling Ph Diagr Thermochem. 2009;33(2):279. doi: 10.1016/j.calphad.2008.09.015

[40] Su C, Xi M, Dai Z-G, et al. Dry etching of GaAs with Cl_2_: correlation between the surface Cl coverage and the etching rate at steady state. Surf Sci Lett. 1993;282(3):357. doi: 10.1016/0167-2584(93)91079-4

[41] Yamaguchi H, Kuramata A, Masui T. Slip system analysis and X-ray topographic study on β-Ga_2_O_3_. Superlatt Microstruct. 2016;99:99. doi: 10.1016/j.spmi.2016.04.030

[42] Mu S, Wang M, Peelaers H, et al. First-principles surface energies for monoclinic Ga_2_O_3_ and Al_2_O_3_ and consequences for cracking of (Al*_x_*Ga_1-*x*_)_2_O_3_. APL Mater. 2020;8(9). doi: 10.1063/5.0019915

