# Supplementary figures and images for: HCl-gas etching behavior of (001) β-Ga2O3 under oxygen supply
Source: Sci Technol Adv Mater. 2025 Sep 3;26(1):2546285. doi: 10.1080/14686996.2025.2546285 (PMC12409918; doi:10.1080/14686996.2025.2546285)

## Slide 1
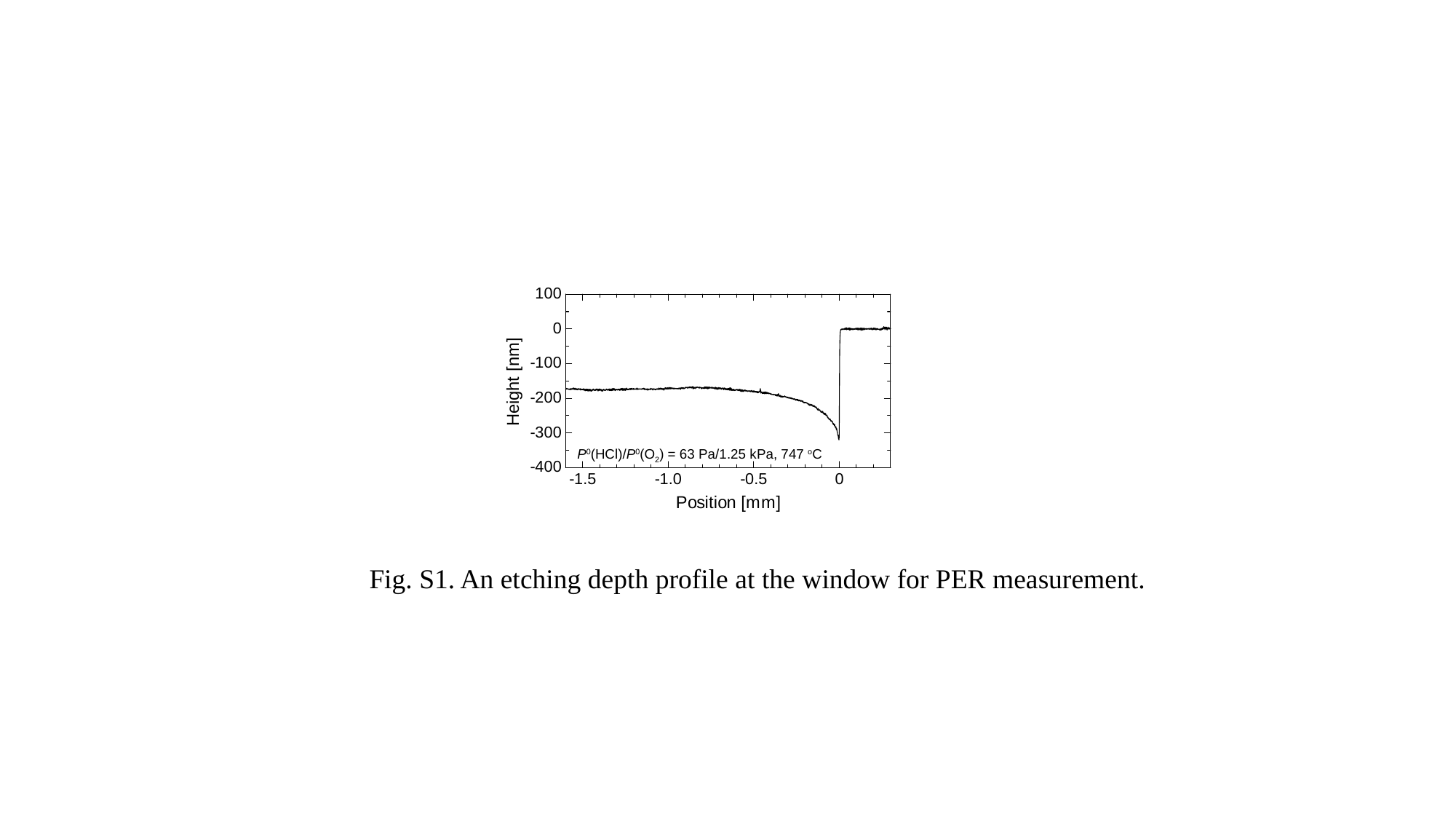

P0(HCl)/P0(O2) = 63 Pa/1.25 kPa, 747 oC
Fig. S1. An etching depth profile at the window for PER measurement.

Supplement: Supplemental Material [file TSTA_A_2546285_SM4930.pptx]
